# Algorithms in Allergy: Hereditary Angioedema

**DOI:** 10.1111/all.70241

**Published:** 2026-02-02

**Authors:** Konrad Bork, Markus Magerl, Emel Aygören‐Pürsün, Henriette Farkas

**Affiliations:** ^1^ Department of Dermatology, University Medical Center Johannes Gutenberg University Mainz Germany; ^2^ Institute of Allergology Charité—Universitätsmedizin Berlin Berlin Germany; ^3^ Immunology and Allergology Fraunhofer Institute for Translational Medicine and Pharmacology ITMP Berlin Germany; ^4^ Department for Children and Adolescents Goethe‐University Frankfurt Frankfurt Germany; ^5^ Hungarian Angioedema Centre of Reference and Excellence, Department of Internal Medicine and Haematology Semmelweis University Budapest Hungary

**Keywords:** angioedema, endotypes, genetics

**Abbreviations**
ACARE: Angioedema Centers of Reference and Excellence
*ANGPT‐1*
: gene encoding angiopoietin‐1
C1INH: C1‐inhibitor
*CPN1*
: gene encoding carboxypeptidase N
*DAB2IP*
: gene encoding disabled homolog 2‐interacting protein
*F12*
: gene encoding coagulation factor XII
FXII: coagulation factor XII
HAE: hereditary angioedema
HAE‐C1INH: hereditary angioedema due to C1‐inhibitor deficiency
HAE‐FXII: hereditary angioedema due to F12 mutations
HAE‐nC1INH: hereditary angioedema with normal C1‐inhibitor
HAE‐PLG: hereditary angioedema due to PLG mutation
*HS3ST6*
: gene encoding heparan sulfate 3‐O‐sulfotransferase 6
*KNG1*
: gene encoding kininogen 1
*MYOF*
: gene encoding myoferlin
pd: plasma‐derived
*PLG*
: gene encoding plasminogen
rh: recombinant human
*SERPING1*
: gene encoding C1‐inhibitor

Angioedema is a clinical sign of various hereditary and acquired conditions (Supporting Information) [1, 2]. The most common type of hereditary angioedema (HAE) results from a genetic C1INH deficiency (HAE‐C1INH) leading to a low plasma activity of C1INH, which is considered to be a rare disease with a prevalence of approximately 1:50,000. It is classified as HAE‐C1INH type I with low C1INH protein concentration (80% of the cases) and in type 2 with normal or elevated C1INH protein concentration in plasma (20% of the cases).

In 2000, a novel type of HAE without wheals was identified [3, 4]. This type was not associated with a deficiency of C1INH and was termed “HAE with normal C1INH” (HAE‐nC1INH or HAE type III). In 2006, missense mutations in the coagulation factor XII gene (*F12*) were identified as the underlying genetic defect [5]. Between 2018 and today (2025), further types of HAE‐nC1INH were detected. Currently, gene mutations in six different genes (*F12*, *PLG*, *ANGPT‐1*, *KNG1*, *MYOF*, *HS3ST6*) linked with the expression of HAE‐nC1INH have been reported. Another two gene variants in *CPN1* and *DAB2IP* are candidate genes under investigation due to their co‐expression with urticaria. HAE–FXII and HAE due to mutations in the plasminogen gene (HAE‐PLG) seem to be the most common types of HAE‐nC1INH; however, these also occur much less frequently than HAE‐C1INH. In a major number of families with HAE‐nC1INH, a basic genetic defect has not yet been identified up to now (HAE‐unknown; HAE‐UNK) [1, 2].

Clinical symptoms of HAE may include swelling of the skin and tongue, abdominal pain attacks, as well as pharyngeal and laryngeal edema that can lead to asphyxiation [6]. HAE‐associated angioedema usually does not occur with urticaria, nor does it respond to high‐dose anti‐allergic medications.

There are a number of clinical conditions (“masqueraders”) that are occasionally mistaken for angioedema. Clinical classification of the various forms of angioedema begins with noting the presence or absence of recurrent wheals as a sign of urticaria [1]. This allows many, but by no means all, cases of recurrent angioedema to be clinically ruled out as chronic spontaneous urticaria. Diagnosis of HAE can be established based on the family history, clinical symptoms, complement and genetic testing (Figure 1, Supporting Information).

Treatment goals encompass preventing asphyxiation, preventing attacks, and controlling disease activity (frequency, severity, and duration of attacks) and enhancing the quality of life (Supporting Information) [7, 8].

Due to the severe symptoms (gastrointestinal attacks, risk of asphyxiation), managing and treating patients with HAE represents a considerable responsibility. It is mandatory to provide patients with comprehensive and detailed information on symptoms, in particular initial symptoms of laryngeal edema, as well as trigger factors. Family screening of first‐degree relatives is obligatory. Patients and their families need a clear plan for managing symptoms. This includes educating family members about the disease and necessary interventions. Patients should also carry an emergency medical ID (identity) card. Given the importance and time‐intensive nature of these measures, it is recommended to refer patients to an HAE treatment center, such as the Angioedema Center of Reference and Excellence (ACARE) to leverage their extensive experience [9]. Ideally, patients are treated close to where they live by their family physician or treating physician in close cooperation with an HAE treatment center. Patients should also be referred to patient self‐help organizations, which make a significant contribution to the care of patients with HAE.

For treating patients with HAE‐C1INH, there is a number of highly effective and safe drugs available (Supporting Information). They include drugs for treating attacks, for short‐term prophylaxis, and for preventing attacks (long‐term prophylaxis) (Tables 1 and 2) [7, 8]. Drugs for treatment of attacks include icatibant (bradykinin B2 receptor antagonist), human plasma‐derived (pd) or recombinant human (rh) C1INH, and the plasma kallikrein‐inhibitors sebetralstat and ecallantide (ecallantide is not licensed for Europe). For long‐term prophylaxis in HAE‐C1INH, the recommended drugs include pdC1INH, the kallikrein inhibitors lanadelumab and berotralstat, pre‐kallikrein inhibitor donidalorsen and the activated FXII blocker garadacimab. Androgens and antifibrinolytics are not considered first‐line drugs for this treatment.

Treatment experience in patients with one of the various types of HAE‐nC1INH is limited, mainly due to the rarity of these conditions [2]. Icatibant and pd. C1INH have been reported to be efficacious in many patients with HAE‐FXII and HAE‐PLG although a significant number of patients reported about little benefit. A similarly inconsistent pattern can be seen in long‐term prophylaxis. While modern therapies for long‐term prophylaxis in HAE‐C1INH have a good to very good efficacy profile, the number of partial or non‐responders in all types of HAE‐nC1INH is comparably high.

**FIGURE 1:**
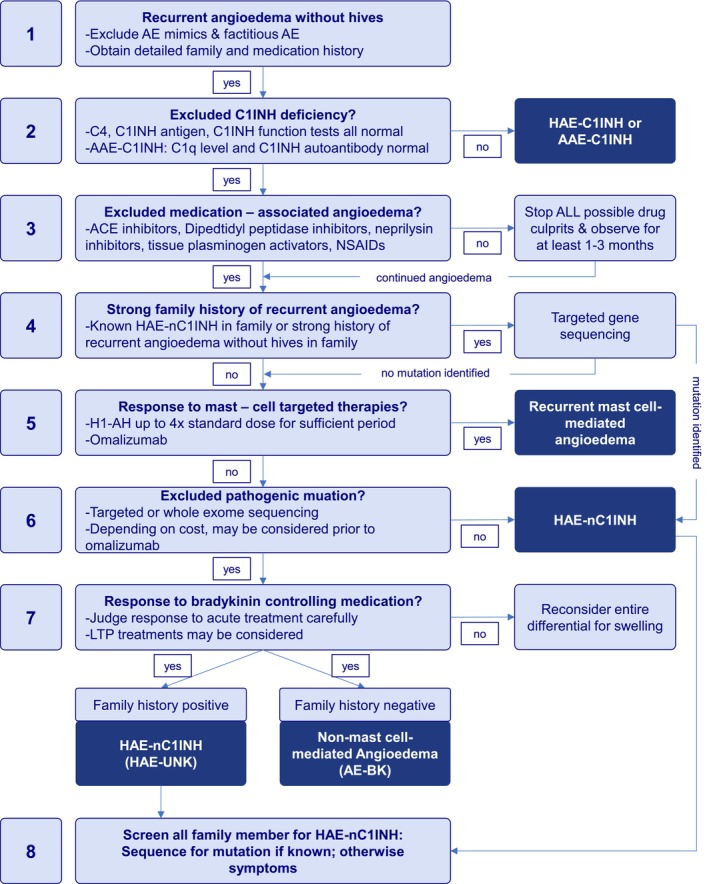
Algorithm for diagnosis of hereditary angioedema. Adapted from Zuraw et al. [2]. Algorithm with the most important steps for the diagnosis of hereditary angioedema. These steps do not necessarily have to be carried out in the chronology shown. Step 1: In the vast majority of cases of mast cell‐mediated angioedema, wheals also occur sporadically, not necessarily at the same time as the angioedema. The anamnestic exclusion of mast cell‐mediated angioedema and other differential diagnoses is the first step. Step 2: HAE‐C1INH and AAE‐C1INH can be diagnosed by determining C1INH and C4 complement, and possibly also C1q and antibodies against C1INH If there is reasonable suspicion of an AAE‐C1INH, C1q levels and antibodies against C1 inhibitor should be determined during the initial consultation. This suspicion is particularly strong if the patient developed the first symptoms after the age of 40 and there is no family history of the disease. Step 3: If medication is being taken that could be responsible for the occurrence of angioedema, it should be discontinued. Step 4: A positive family history of angioedema should be clarified by molecular genetic testing. Step 5 and Step 6: In the absence of a positive family history or in the absence of a causative mutation in the molecular genetic test, a therapy trial with antihistamines (H1‐AH) or better omalizumab can show whether it is a case of mast cell‐mediated angioedema without accompanying wheal formation. A sufficiently long prophylactic therapy is recommended; in the case of antihistamines, the dose should exceed the standard dose recommendation by the fourfold (i.e., in case of cetirizin 40 mg per day). The response to acute treatment with antihistamines, glucocorticoids, or epinephrine is more difficult to assess than the response to prophylactic treatment, but it can also provide indications of the presence of mast cell‐mediated angioedema. Step 7: Application ex juvantibus of drugs that control the excessive formation of bradykinin. Particularly in the case of on‐demand treatment, very thorough documentation of the course of symptoms after treatment and cautious interpretation of positive results is recommended. With long‐term prophylaxis, it is much easier to assess the effect of the therapy, as the frequency and severity of attacks are easier to measure than the timing of symptom relief after on‐demand treatment. The indication for long‐term prophylaxis is generally based on the recommendations of the international HAE guideline, although different criteria must be applied, at least temporarily, for the use of ex juvantibus therapy to make a diagnosis. If the symptoms clearly respond to medication that controls bradykinin formation, an HAE‐nC1INH of unknown cause can be diagnosed if there is a positive family history of angioedema, and a non‐mast cell‐mediated angioedema of unknown cause if there is no family history. Step 8: If HAE‐nC1INH has been diagnosed, family members should also undergo diagnostic procedures. Cascade screening of potentially affected family members should also be performed on clinically unremarkable individuals, as asymptomatic carriers of the disease are known to exist, particularly in the case of HAE‐nC1INH. AAE‐C1INH, acquired C1INH‐ deficiency; ACE, angiotensin converting enzyme; AE, angioedema; C1INH, C1‐inhibitor; C1q, complement component 1; H1‐AH, H1‐antihistamine; HAE, hereditary angioedema; HAE‐C1INH, hereditary angioedema with C1‐inhibitor deficiency; HAE‐nC1INH, hereditary angioedema with normal C1‐inhibitor; HAE‐UNK, HAE‐unknown; LTP, long‐term prophylaxis; NSAIDs, non‐steroidal anti‐inflammatory drugs.

**TABLE 1:** Currently available drugs for acute treatment of HAE attacks in HAE‐C1INH.

Mode of action	Drug class	Age groups		Mode of administration	
		Children	Adolescents/adults		
Replacement of lacking protein	Plasma‐derived C1‐INH	CSL: “Pediatric” No age limit Takeda: No ≥ 2 years	CSL: Yes No age limit Takeda: No Yes	IV	Self‐administration
	Recombinant C1‐INH	No ≥ 2 years	Yes Yes		
Antagonism of the bradykinin B2 receptor	Icatibant	No ≥ 2 years	No (≥ 18 years) Yes	SC	
Inhibition of kallikrein	Ecallantide	No No	≥ 12 years No	SC	By healthcare professional
	Sebetralstat	No No	≥ 12 years ≥ 12 years	Oral	Self‐administration

*Note:* Fresh frozen plasma/Solvent detergent plasma are second‐line therapies. (1) FDA‐Approved Drugs (Drugs@FDA). https://www.accessdata.fda.gov/scripts/cder/daf/ (last accessed September 28, 2025). (2) FDA Approved Blood Products. https://www.fda.gov/vaccines‐blood‐biologics/blood‐blood‐products/approved‐blood‐products (last accessed September 28, 2025). (3) European Union Register of Medicinal Products. https://ec.europa.eu/health/documents/community‐register/html/reg_hum_act.htm?sort=a (last accessed September 28, 2025).

Abbreviations: IV, intravenous; SC, subcutanous.

**TABLE 2:** Currently available drugs for long‐term prophylaxis in HAE C1INH.

Mode of action	Drug class	Pediatric age groups		Mode of administration	
		Children	Adolescents		
Replacement of lacking protein	IV plasma‐derived C1‐INH	Takeda: ≥ 6 years ≥ 6 years	Takeda: Yes Yes	IV	Self‐administration
	SC plasma‐derived C1‐INH	CSL: ≥ 6 years No	CSL: Yes Yes	SC	
Inhibition of kallikrein	Lanadelumab	≥ 2 years ≥ 2 years	Yes Yes	SC	
	Berotralstat	No No	≥ 12 years ≥ 12 years	Oral	
Prekallikrein‐directed antisense oligonucleotide	Donidalorsen	No (no license yet)	≥ 12 years (no license yet)	SC	
Inhibition of FXIIa	Garadacimab	No No	≥ 12 years ≥ 12 years	SC	
**Non‐targeted 2nd‐line therapies**					
Unknown precisely	Attenuated androgens	No	Yes/No^a^	Oral	
Inhibition of plasminogen	Antifibrinolytics	Yes/No^a^	Yes/No^a^	Oral	

*Note:* (1) FDA‐Approved Drugs (Drugs@FDA). https://www.accessdata.fda.gov/scripts/cder/daf/ (last accessed September 28, 2025). (2) FDA Approved Blood Products. https://www.fda.gov/vaccines‐blood‐biologics/blood‐blood‐products/approved‐blood‐products (last accessed September 28, 2025). (3) European Union Register of Medicinal Products. https://ec.europa.eu/health/documents/community‐register/html/reg_hum_act.htm?sort=a (last accessed September, 2025).

^a^ Age isNot specified in the prescribing information. Indications may vary according to countries and according to manufacturers.

## Author Contributions

K.B. conceptualized the manuscript, literature review and material preparation; M.M. writing: material preparation, review and editing; E.A.‐P. writing: material preparation, review and editing; H.F. conceptualized the manuscript, literature review and material preparation.

## Funding

The authors have nothing to report.

## Conflicts of Interest

K.B. has received honoraria and/or research funding, and/or has served as a consultant and/or participated in advisory boards for CSL Behring, Pharvaris, and Takeda. M.M. has served as an advisor/consultant/speaker for Argo, Astria, BioCryst, CSL Behring, Intellia Therapeutics, KalVista Pharmaceuticals, Otsuka, Pharvaris, and Takeda, and been a speaker for CSL Behring, Pharming, and Takeda. E.A.‐P. has served as an advisor/consultant/speaker for Astria, BioCryst, Biomarin Europe, CSL Behring, Intellia Therapeutics, KalVista Pharmaceuticals, Otsuka, Pharvaris, and Takeda, and has been a speaker for Biocryst, CSL Behring, Pharming, and Takeda. H.F. has received research grant from CSL Behring and Pharvaris; speaker fee from Biocryst, CSL Behring, Pharming, Pharvaris, and Takeda, travel grants from Astria, Biocryst, CSL Behring, Intellia, Ionis, KalVista, ONO Pharmaceutical, Pharming, Pharvaris, and Takeda; served as an advisor/consultant for Astria, Biocryst, CSL Behring, Intellia, Ionis, KalVista, ONO Pharmaceutical, Pharming, Pharvaris, and Takeda.

## Supporting information


**Data S1:** all70241‐sup‐0001‐Supinfo.docx.

## Data Availability

No data was generated for this article.
