# Supplementary material for: Algorithms in Allergy: Hereditary Angioedema
Source: Allergy. 2026 Feb 2;81(8):3008–12. doi: 10.1111/all.70241 (PMC13466138; doi:10.1111/all.70241)
Supplement: Supplementary file 1 [file ALL-81-3008-s001.docx]

**Supporting Information**

**Pathogenesis**

Pathogenesis of angioedema can be considered in two broad categories: Mast cell mediated with release of vasoactive mediators; and bradykinin driven where increased vascular permeability is mediated by bradykinin. Mast cell-mediated angioedema becomes a challenge as a differential diagnosis to hereditary angioedema, especially when it occurs as recurrent isolated angioedema without a history of wheals. In about 10% of cases of chronic spontaneous urticaria, only isolated angioedema occurs.

The most common type of hereditary angioedema (HAE) results from a genetic C1INH deficiency (HAE-C1INH) leading to decreased production of C1INH and is classified as HAE-C1INH -Type 1) [1].Variants in *SERPING1* may result in decreased C1INH activity while C1INH serum levels remain normal, causing HAE-C1INH-Type 2. [2] During angioedema attacks in HAE-C1INH, the contact-kallikrein-kinin system is activated, resulting in an overproduction of the vasoactive peptide bradykinin.

Gene mutations in HAE with normal C1-INH: HAE with *F12* gene pathogenic variants (HAE–FXII) and HAE due to plasminogen mutation (HAE-PLG) seem to be the most common types of HAE-nC1INH. Up to now, the other types of HAE-nC1INH with identified basic variants have been reported in one family or in very few families only [3-5].

The pattern of affected family members suggests an autosomal dominant mode of inheritance both in HAE-C1INH and HAE-nC1INH. In the latter type there is incomplete penetrance with more females affected.

**Diagnosis**

The diagnosis of HAE requires complement testing. With complement testing the different types of C1INH deficiency can be confirmed or excluded. Genetic testing is usually not required for the diagnosis of HAE-C1INH because the disease can be well diagnosed with a complement test. The diagnosis of HAE-nC1INH is more challenging: Once that mast cell-mediated angioedema and C1INH-deficiency (HAE-C1INH) have been ruled out, the focus for the diagnosis of HAE-nC1INH is on the detection of genetic and familial clues. The decisive step towards a definitive diagnosis is molecular genetic analysis. Ideally, this should include all known causative mutations. If molecular genetic testing fails to identify a causative mutation, a clear family history is indispensable [5, 6]. It is worth mentioning that a positive family history is the rule in hereditary angioedema, but especially in HAE-C1INH there are exceptions to this rule due to spontaneous mutations (approximately 20% of cases). On the other hand, a family history does not completely rule out mast cell mediated angioedema. A few cases of familial chronic spontaneous urticaria have been reported in the literature.

**Management of patients with HAE**

Regular patient information sessions are crucial for effective patient management and should be meticulously documented. It is imperative that these sessions provide patients with comprehensive and detailed information regarding their condition. This includes, but is not limited to, a thorough explanation of all potential symptoms, with particular emphasis on the initial signs of critical conditions such as laryngeal edema. Furthermore, patients must be educated about specific trigger factors that could exacerbate their condition or lead to adverse events. Beyond symptom and trigger factor awareness, each patient should receive a personalized action plan, which should be individualized through shared decision-making, considering patient preferences and values. This plan will outline precise steps to take in response to the onset of symptoms, especially in emergency situations. This empowers patients to take an active role in their self-management and can significantly improve outcomes. Treatment plans should be individualized through shared decision-making, considering patient preferences and values Moreover, patients should be actively involved in and educated on self-administering treatments. All aspects of these information sessions, including the content discussed, the patient's understanding, and the provision of the individualized plan, must be thoroughly recorded in their medical file for future reference and continuity of care. Likewise, family members should be informed about the disease and the necessary measures and family screening to be taken.

Treatment strategy consists of acute treatment of HAE attacks and prophylaxis [5, 7-9].

**Drug treatment**

Treatment for HAE attacks should be administered as soon as possible to maximize efficacy. Acute treatment aims at minimizing symptoms and risk of suffocation, drugs for acute treatment include intravenous pdC1INH and rhC1INH concentrates, the subcutaneous B2-recptor antagonist icatibant and the oral kallikrein-inhibitor sebetralstat).

Short-term prophylaxis with pdC1INH is considered before medical, surgical, or dental procedures as well as exposure to other angioedema attack-inducing events. Fresh frozen or solvent detergent plasma can be used for acute treatment and short-term prophylaxis if first line treatments are not available [10, 11].

Drugs used for long-term prophylaxis are intravenous and subcutaneous pdC1INH, the kallikrein-inhibitor lanadelumab (monoclonal antibody), berotralstat (small molecule), the activated FXII inhibitor garadacimab (monoclonal antibody) and donidalorsen (prekallikrein-directed antisense oligonucleotide) [12, 13]. Attenuated androgens and antifibrinolytics do not belong to the first-line drugs recommended for long-term prophylactic treatment in HAE-C1INH [14].

**HAE-nC1INH**

The evidence base for the treatment of HAE-nC1INH is currently limited and is based mainly on expert opinions, case reports, and small case series, as there is a lack of controlled clinical trials. Much of the available treatment data comes from the HAE-nC1INH Consensus Treatment (HCT) Survey, a survey of global experts that compiled information on 594 patients from 15 countries. This survey provided expert ratings of the effectiveness of various therapies on a Likert scale [5]. Since HAE-nC1INH is a group of disorders with similar clinical symptoms but different pathogenesis, it was not unexpected that response rates to treatment vary depending on the subtype. In a recent controlled study which contained 5 patients with a known HAE-nC1INH specific mutation and 13 patients with HAE-unknown the difference in attack number reduction between the lanadelumab and the placebo group did not reach significance (although the attack number was lower in the lanadelumab group) [15]. In another recently published study with garadacimab, the uncontrolled monthly administration of 200 mg s.c. was shown to be partially effective. Garadacimab demonstrated efficacy in 2 out of 3 patients with HAE-FXII (including absence of attacks in the extension phase). In HAE-PLG, however, a reduction in the attack rate was only observed in one out of three patients.[16].

References

1.	Donaldson, V.H. and R.R. Evans, *A Biochemical Abnormality in Herediatry Angioneurotic Edema: Absence of Serum Inhibitor of C' 1-Esterase.* Am J Med, 1963. **35**: p. 37–44.

2.	Rosen, F.S., et al., *Hereditary Angioneurotic Edema: Two Genetic Variants.* Science, 1965. **148**(3672): p. 957–8.

3.	Christiansen, S.C., et al., *Hereditary Angioedema With Normal C1 Inhibitor: A Quarter Century of Forward Progress and Persisting Obstacles.* J Allergy Clin Immunol Pract, 2025. **13**(6): p. 1300–1309.

4.	Magerl, M., et al., *Global frequency, diagnosis, and treatment of hereditary angioedema with normal C1 inhibitor.* J Allergy Clin Immunol Glob, 2025. **4**(3): p. 100446.

5.	Zuraw, B.L., et al., *Hereditary Angioedema with Normal C1 Inhibitor: an Updated International Consensus Paper on Diagnosis, Pathophysiology, and Treatment.* Clin Rev Allergy Immunol, 2025. **68**(1): p. 24.

6.	Buttgereit, T., et al., *Case report: Recurrent angioedema: Diagnosing the rare and the frequent.* Front Med (Lausanne), 2022. **9**: p. 1048480.

7.	Betschel, S., et al., *The International/Canadian Hereditary Angioedema Guideline.* Allergy Asthma Clin Immunol, 2019. **15**: p. 72.

8.	Maurer, M., et al., *The international WAO/EAACI guideline for the management of hereditary angioedema-The 2021 revision and update.* Allergy, 2022. **77**(7): p. 1961–1990.

9.	Riedl, M.A., et al., *Shared decision-making in the management of hereditary angioedema: An analysis of patient and physician perspectives.* Allergy Asthma Proc, 2022. **43**(5): p. 397–405.

10.	Farkas, H., et al., *Short-term prophylaxis in hereditary angioedema due to deficiency of the C1-inhibitor--a long-term survey.* Allergy, 2012. **67**(12): p. 1586–93.

11.	Magerl, M., et al., *Short-term prophylactic use of C1-inhibitor concentrate in hereditary angioedema: Findings from an international patient registry.* Ann Allergy Asthma Immunol, 2017. **118**(1): p. 110–112.

12.	Fasshauer, M. and B. Wedi, *Hereditary angioedema (HAE) in children and adolescents: New treatment options.* Allergol Select, 2024. **8**: p. 336–345.

13.	Recke, A., *Status quo and future developments in the diagnosis and treatment of hereditary angioedema.* J Dtsch Dermatol Ges, 2025.

14.	Maurer, M., et al., *Attenuated androgen discontinuation in patients with hereditary angioedema: a commented case series.* Allergy Asthma Clin Immunol, 2022. **18**(1): p. 4.

15.	Riedl, M.A., et al., *Lanadelumab for prevention of attacks of non-histaminergic normal C1 inhibitor angioedema: results from the randomized, double-blind CASPIAN Study and CASPIAN open-label extension.* Front Immunol, 2025. **16**: p. 1502325.

16.	Cohn, D.M., et al., *Garadacimab in hereditary angioedema due to normal C1INH with F12/PLG mutations.* J Allergy Clin Immunol, 2025.
